# Toughened Poly (Lactic Acid)—PLA Formulations by Binary Blends with Poly(Butylene Succinate-*co*-Adipate)—PBSA and Their Shape Memory Behaviour

**DOI:** 10.3390/ma12040622

**Published:** 2019-02-19

**Authors:** Diego Lascano, Luis Quiles-Carrillo, Rafael Balart, Teodomiro Boronat, Nestor Montanes

**Affiliations:** 1 Escuela Politécnica Nacional, 17-01-2759 Quito, Ecuador; dielas@epsa.upv.es; 2 Technological Institute of Materials (ITM), Universitat Politècnica de València (UPV), Plaza Ferrándiz y Carbonell 1, 03801 Alcoy, Spain; rbalart@mcm.upv.es (R.B.); tboronat@dimm.upv.es (T.B.); nesmonmu@upvnet.upv.es (N.M.)

**Keywords:** poly (lactic acid) (PLA), poly(butylene succinate-co-adipate) (PBSA), binary blends, shape memory behaviour

## Abstract

This study reports the effect of poly(butylene succinate-*co*-adipate) (PBSA) on the mechanical performance and shape memory behavior of poly(lactic acid) (PLA) specimens that were manufactured by injection molding and hot-press molding. The poor miscibility between PLA and PBSA was minimized by the addition of an epoxy styrene-acrylic oligomer (ESAO), which was commercially named Joncryl^®^. It was incorporated during the extrusion process. Tensile, impact strength, and hardness tests were carried out following international standards. PLA/PBSA blends with improved mechanical properties were obtained, which highlighted the sample that was compatibilized with ESAO, leading to a remarkable enhancement in elongation at break, but showing poor shape memory behaviour. Field Emission Scanning Electron Microscopy (FESEM) images showed how the ductile properties were improved, while PBSA loading increased, thus leading to minimizing the brittleness of neat PLA. The differential scanning calorimetry (DSC) analysis revealed the low miscibility between these two polymers and the improving effect of PBSA in PLA crystallization. The bending test carried out on the sheets of PLA/PBSA blends showed the direct influence that the PBSA has on the reduction of the shape memory that is intrinsically offered by neat PLA.

## 1. Introduction

The use of polymers and plastics in our daily life is almost mandatory due to their huge range of properties. For this reason, the demand for these materials has remarkably increased in the last decades. Unlike their production, the treatment of these materials after their end-of-life has been neglected, resulting in the oversaturation of plastic wastes in the environment. Since most of these plastics are synthetic, petroleum-derived materials, they have a high resistance to microbial degradation, so their decomposition is complex and extremely slow [1]. The development of new environmentally friendly polymeric materials has become a leading force in this industry because of this. The environmentally friendly properties of a polymer could be related to their origin (bio-sourced) or to their end-of-life (biodegradable or disintegrable in controlled compost soil). Taking into account their origin, some of the polymers have been fully or partially obtained from renewable resources, such as poly(ethylene) (PE) and poly(propylene) (PP) from sugarcane, poly(amides) (PAs) from castor oil, poly(carbonate) (PC) from corn, and so on [2,3,4]. These biobased polymers are identical to their counterpart petroleum-derived polymers and despite that they are not biodegradable, they have a positive effect on carbon footprint [3]. Another interesting group of environmentally friendly polymers is that which includes some petroleum-derived poly(esters), but, due to the nature of the ester group, they can disintegrate in controlled compost conditions. This groups includes poly(ε-caprolactone) (PCL), poly(butylene succinate) (PBS), poly(butylene succinate-co-adipate) (PBSA), poly(glycolic acid) (PGA), among others [1,5,6,7]. Finally, the most interesting group of environmentally friendly polymers is that of biobased and biodegradable polymers. Polysaccharides (starch, cellulose, chitin, and so on), protein based polymers (gluten, soybean protein, casein, collagen, among others), and bacterial polymers, such as poly(3-hydroxybutyrate) (PHB) or poly(3-hydroxybutyrate-*co*-3-hydroxyvalerate) (PHBV) are included in this group. Although these polymers are very promising, their properties are still quite far from those of commodities and engineering plastics [8,9,10,11].

Nowadays, poly(lactic acid) (PLA) is one of the most studied aliphatic polyesters thanks to its good mechanical properties and it can be either obtained from petroleum or renewable resources. Bio-sourced PLA is produced by anaerobic fermentation of sugars that are derived starch-rich plants, such as corn, sugarcane, beet sugar, potato, and so on [12]. It can also be obtained through the direct condensation of lactic acid and by ring opening polymerization of cyclic lactide dimer (ROP) [6,13]. The excellent tensile strength that PLA presents is the reason why it is used as an alternative to conventional plastics, such as high and low-density poly(ethylenes) (HDPE/LDPE), poly(styrene) (PS), and poly(ethylene terephthalate) (PET) [6]. It can be manufactured in a wide range of processing methods, such as conventional extrusion, injection molding, blow molding, film forming, three-dimensional (3D)-printed, and so on [14,15,16,17]. All of these features make PLA exceptionally useful in the packaging industry, food containers [12,18], and in biomedicine for controlled drug delivery and tissue engineering [19]. Although PLA presents good balanced properties and remarkable environmental benefits, its use has been limited due to its cost [13] and, in addition, it is a quite brittle material. With the aim of improving the ductility and toughness of PLA [20], many research approaches have been used. One is plasticization with conventional plasticizers, such as poly(ethylene glycol) (PEG), poly(propylene glycol) (PPG), lactic acid oligomers (OLAs), modified vegetable oils (MVOs), esters from citric acid or adipic acid, and so on [21,22,23]. All of these plasticizers contribute to improving ductile properties due to a remarkable decrease in the glass transition temperature, T_g_, but the mechanical resistant properties are highly reduced. Another approach to overcome (or minimize) the extremely high brittleness of PLA is by blending with other flexible polymers. Regarding blends, compatibility/miscibility issues must be taken into account [24]. Bhatia et al. [25] have reported the properties of binary blends of PLA with PBS. The addition of 30 wt % PBS to PLA resulted in a clear increase in toughness, but over 50 wt % PBS addition, the clarity of the materials is reduced. As PLA and PBS show restricted miscibility, Harada et al. [26] used an isocyanate as a reactive processing aid to increase the impact strength of PLA/PBS binary blends. PCL is another flexible aliphatic poly(ester) that can provide increased toughness to PLA, as reported by Matta et al. [6]. Poly(butylene adipate-*co*-terephthalate) (PBAT) has great flexibility and it maintains excellent biodegradability properties. As can be shown by Khatsee et al. [27], binary PLA/PBAT blends can be obtained by electrospinning for controlled antibiotic release. Poly(propylene carbonate) (PPC) has been successfully used in PLA blends with good shape memory polymers [28]. 

Taking advantage of the PLA structure and materials that are based on PLA, a new research field has emerged, which is shape memory. Because of its particular structure, this field is promising for PLA. PLA has crystalline domains that define the permanent shape and switching segments that fix the temporary shape [29]. This particular structure allows for PLA to switch from a temporary shape to its permanent shape. The switching segments are activated by an external stimulus that can be either physical or chemical, but temperature is the most common stimulus leading to a thermal-responsive memory shape by selecting the appropriate temperature cycle regarding the T_g_ [30,31,32]. Shape memory behaviour has been used in electronics [29], and one of most recent applications include pieces that are made by 3D printing with PLA matrix filaments [33]. As these 3D-printed parts can change their shape by heating/cooling above/below the T_g_, this technology is known as four-dimensional (4D)-printing. Taking advantage of the PLA biocompatibility with the human body, recent applications of PLA are focused on tissue engineering by making scaffold systems of physical blends, with TPU for support material in cartilage or bone repair [32,34]. The aim of this research is to enhance PLA toughness, through physical blending with poly(butylene succinate-*co*-adipate) (PBSA) to obtain flexible shape memory polymers with improved toughness. Despite that PBSA is based on fossil resources, it can undergo disintegration in controlled compost [35,36]. PBSA could offer high flexibility and great impact strength properties to PLA [37]. As these two poly(esters) show restricted miscibility, an epoxy styrene-acrylic oligomer (ESAO) will be used.

## 2. Materials and Methods 

### 2.1. Materials.

PLA was an IngeoTM Biopolymer 6201D that was obtained from NatureWorks (Minnetonka, MN, USA). It is a thermoplastic resin derived from annually renewable resources. Its glass transition temperature, T_g_, is comprised between 55–60 °C and the melt peak temperature is located in the 155–170 °C range. Its density is 1.25 g cm^−3^ and it possesses a melt flow index, MFI of 15–30 g/10 min. With regard to the flexible polymer for binary blends, an aliphatic poly(ester) copolymer, PBSA, was used. A commercial Bionolle grade 3002 MD was obtained from Showa Denko Europe GmbH (Munich, Germany). It shows good processability for extrusion and blow molding. The density reported is for this PBSA grade is 1.23 g cm^−3^. Regarding its thermal properties, its T_g_ is close to −45 °C and the melt peak temperature is 94 °C.

**Figure 1:**
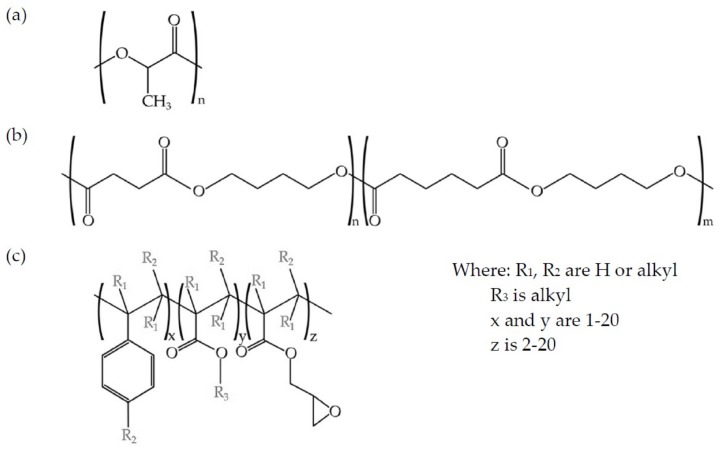
Schematic representation of (**a**) poly(lactic acid) (PLA), (**b**) poly(butylene succinate-*co*-adipate) (PBSA), and (**c**) epoxy styrene-acrylic oligomer (ESAO) (Joncryl^®^ ADR-4300).

A multi-functional epoxy-based styrene-acrylic oligomer (ESAO) was used as a compatibilizer. A commercial ESAO grade JONCRYL^®^ ADR-4300 that was distributed by Basf (Barcelona, Spain) was used. This compatibilizer has a glass temperature T_g_ of 56 °C and an epoxy equivalent weight of 445 g mol^−1^. Figure 1 shows the schematic representation of the structures of both PLA and PBS and the ESAO generic structure.

### 2.2. Manufacturing of PLA/PBSA Binary Blends

Prior to processing, as both of the poly(esters) are highly sensitive to hydrolysis, PLA and PBSA were dried at 50 °C for 48 h. Table 1 shows the labelling and the compositions of the developed blends. According to the literature [38], PLA blends with 20 wt % of PBS have a good balance between mechanical properties and shape memory behaviour. Thus, the addition of a compatibilizer to the PLA_80_PBSA_20_ mixture was decided to determine whether it causes any effect in its properties. To obtain homogeneous mixtures, all of the materials were subjected to a mechanical pre-mixing for 3 min in a zipper bag. These mixtures were extruded using a co-rotating twin-screw extruder ZSK-18 MEGAlab from Coperion (Stuttgart, Germany) that was equipped with a screw diameter of 18 mm with a length to diameter ratio, L/D of 48. The dosage of each component was controlled by a side twin-screw feeder ZS-B 18 from K-Tron (Pitman, NJ, USA). The screw speed was set to 180 rpm using a temperature profile of 145–155–160–180–185–190–190 °C from the feeding to the die. The feed rate was set to 2 kg h^−1^. Once the different blends were extruded, they were cooled down to 15 °C in a water bath and subsequently pelletized using an air knife unit. 

The pelletized compounds were shaped into standard samples for characterization by injection molding in a Meteor 270/75 from Mateu & Solé (Barcelona, Spain). The temperature profile was set at 175–180–185–190 °C for PLA-based blends, while PBSA was programmed with a lower temperature profile of 105 °C for the different heating barrels. A clamping force of 75 tons was applied. The cooling time was set to 10 s.

### 2.3. Mechanical Properties

The tensile test was carried out following the recommendation of the ISO 527 standard while using a mechanical universal testing machine ELIB 50 from S.A.E. Ibertest (Madrid, Spain). It was equipped with a 5 kN load cell and the selected crosshead speed of 10 mm min^-1^ was used. The impact strength test was performed on a Charpy pendulum from Metrotec (San Sebastián, Spain), following the recommendations of ISO 179, using a 6 J pendulum on unnotched samples and a 1 J pendulum on notched samples (“V” type, 2 mm depth and a radio of 0.5 mm). Hardness measurements were obtained in a durometer 676-D from J. Bot Instruments (Barcelona, Spain) using Shore D scale following ISO 868.

All of the tests were carried out at room temperature with at least five samples and the corresponding properties were averaged.

### 2.4. Thermal Characterization

The main thermal transitions were analyzed by differential scanning calorimetry (DSC) in an 821 DSC calorimeter from Mettler-Toledo, Inc. (Schwerzenbach, Switzerland). The samples weight was between 5–10 mg. The samples were placed into standard sealed aluminum pans (40 µL) and then subjected to a thermal cycle consisting of three steps: initial heating from 30 °C to 200 °C, followed by a cooling process to −60 °C and after that, a second heating process up to 350 °C. The heating/cooling rate was set to 10 °C min^−1^. These tests were carried out under a dry atmosphere with a constant nitrogen flow of 30 mL min^−1^. The glass temperature (T_g_), cold crystallization temperature peak (T_cc_), the melting temperature (T_m_), and the melt enthalpy (ΔH_m_) were obtained from second heating step in this analysis.

The thermal degradation (weight loss) and thermal stability were followed by thermogravimetric analysis (TGA) in a TGA/SDTA 851 thermobalance from Mettler-Toledo (Schwerzenbach, Switzerland). The average weight of the samples was between 5–10 mg. These samples were placed on standard alumina crucibles with a total volume capacity of 70 µL and subsequently exposed to a heating program from 30 °C up to 700 °C at a constant heating rate of 20 °C min^−1^ in an air atmosphere. 

### 2.5. Morphology Characterization by Field Emission Scanning Electron Microscopy (FESEM)

To analyze the morphology of fractured surfaces from impact tests, field emission scanning electron microscopy (FESEM) was used. A ZEISS ULTRA 55 FESEM microscope from Oxford Instruments (Abingdon, UK) was used working at an accelerating voltage of 2 kV. Prior to the test, the samples’ surfaces were coated by an ultrathin gold-palladium layer in a high vacuum sputter coater EM MED20 from Leica Microsystem (Milton Keynes, UK).

### 2.6. Shape Memory Behaviour Characterization

The shape memory behaviour of the materials was evaluated using a conventional bending test on sheets with dimensions of 65 × 10 × 1 mm^3^. These sheets were obtained by hot-press molding at 140 °C in a hot press Hoytom M.N.1417 (Bilbao, Spain) from Robima S.A. The switch transition temperature that leads the programming and the recovery cycle was set at 70 °C (PLA glass transition). The cooling temperature was set to 22 °C and the stabilization time was 15 min. The temporary shape was fixed to bending angles of 120° and 90°.

## 3. Results

### 3.1. Mechanical Properties and Morphology of Binary PLA/PBSA Blends

The tensile behaviour of binary PLA/PBSA blends is shown in Table 2. The tensile modulus, E_T_ of neat PLA is relatively high, about 1165 MPa. Regarding its tensile strength (σ_T_), PLA offers a relatively high value of 64.0 MPa, as compared to other commodities. While intrinsic mechanical resistant properties of neat PLA are high, its elongation (ε_b_) at break is only of 9.23%, which is responsible for high brittleness and the fragility of this material. Blending PLA with a flexible PBSA polymer has noticeable effects on overall mechanical performance. So that, the addition of 10 wt % PBSA to PLA leads to an expected decrease on mechanical resistant properties, such as E_T_ and σ_T_, down to values of 1012 MPa and 52.6 MPa, respectively. As the wt % PBSA increases, both tensile modulus and strength decrease, which means that the brittle behaviour is diminished. At room temperature, PBSA is above its corresponding T_g_ value −41 °C [36], which means a flexible behaviour, as observed in Table 2. Above its T_g_, the PBSA chains can move freely, as reported by Ojijo et al. [39], thus leading to high ε_b_ value of 432.7%. This improved chain mobility can exert a positive effect on PLA toughness.

As expected, with PBSA addition, the elongation at break tends to increase. Only the addition of 10 wt % PBSA to PLA gives an increased ε_b_ value of 12.2% and this is still more evident for PLA/PBSA blends containing 20 wt % and 30 wt % PBSA with ε_b_ values of 29.7% and 56.5%, respectively, thus leading to a clear increase in mechanical ductile properties.

Although good mechanical properties can be obtained by blending PLA with PBSA, the poor miscibility between these two polymers does not allow good interface interactions, as reported by Nofar et al. [40]. For this reason, a compatibilizer agent, namely Joncryl^®^, has been added to the binary blend of 80 wt % PLA and 20 wt % PBSA. This compatibilizer agent has been extensively used as chain extender in poly(esters) due to the reaction of epoxy groups with hydroxyl terminal groups in poly(esters) [41,42]. This particular behaviour can be positive in binary blends of poly(esters), as this compatibilizer can react either with the hydroxyl terminal groups of PLA and PBSA, thus leading to a compatibilization effect. The relatively low compatibilizer loading (0.5 phr) allows for this, since higher loadings could lead to branching, gel formation, and, even, some crosslinking [43,44]. As observed in Table 2, the uncompatibilized blend containing 20 wt % PBSA gives an ε_b_ value of 29.7%, while the compatibilized blend with the same composition offers an ε_b_ increase up to values of 121.2%, thus giving clear evidences of chain extension/compatibilization, as reported by Eslami et al. [45]. A low percentage of chain extender in blends leads to obtaining an improvement in the interface with both materials, improving some properties. In this particular case, this blend takes advantages of PBSA, improving the elongation at break. The increase in the ductile behaviour observed in PLA/PBSA blends and, specifically in the compatibilized PLA/PBSA blend, suggest an increase in toughness.

**Figure 2:**
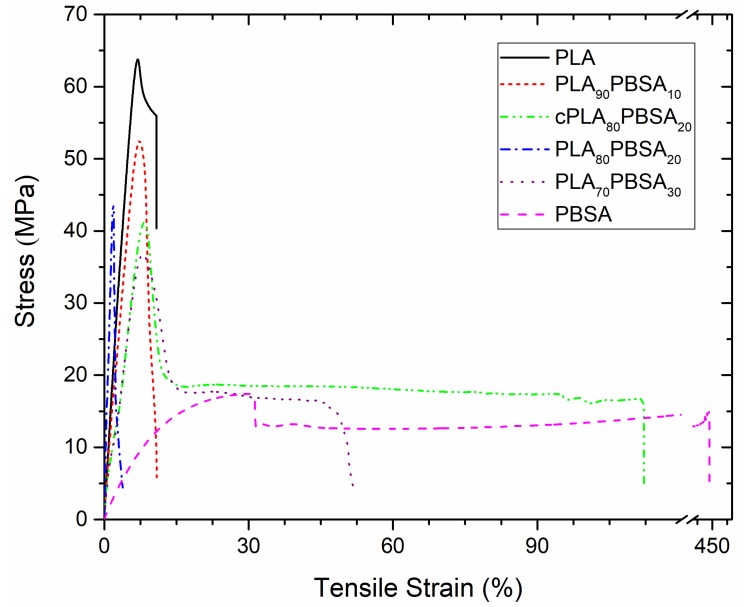
Strain–stress curves corresponding to binary PLA/PBSA blends.

Nevertheless, it is worthy to note that toughness is not uniquely linked to ductile properties (i.e. elongation at break), but also to mechanical resistant properties (tensile strength). In this work, toughness has been quantitatively measured through the determination of the impact-absorbed energy in impact test (Charpy). Table 3 shows the impact strength values as a function of the composition of the developed materials. In a very first attempt, a 6 J pendulum was used, it did not provide enough energy to break some specimens, in order to have all the measurements in the same conditions, a “V” notch was done in all samples and then tested with a 1 J pendulum. As one can see, PLA is a brittle material with very low energy absorption (2.48 kJ m^−2^) when compared to PBSA (26.02 kJ m^−2^). As expected, the impact strength increases with the PBSA loading on binary blends up to values of 5.75 kJ m^−2^ for the uncompatibilized PLA/PBSA blend containing 30 wt % PBSA. It is worthy to note the good impact strength that was achieved with the blend with 20 wt % PBSA (3.28 kJ m^−2^) and the clear positive effect of the compatibilizing effect provided by Joncryl^®^, since the same blend is able to reach an impact strength of about 4.33 kJ m^−2^. Figure 2 presents the stress-strain curves of the developed blends. As it was expected, the stress tends to decrease when PBSA is added. On the other hand, the tensile strain or percentage elongation tends to increase, with the case of the compatibilized blend (cPLA_80_PBSA_20_) being more noticeable, which shows strain values of about 120%. This results in an increase in the area below the curve, together with the increasing tendency of the impact strength, makes an improvement on toughness when PBSA is added. With regard to hardness, the tendency is similar to other mechanical resistant properties. A clear decreasing tendency in the Shore D hardness values can be observed when PBSA is added. Nevertheless, the standard deviation does not allow for identifying a clear effect of the compatibilizer on Shore D values.

**Figure 3:**
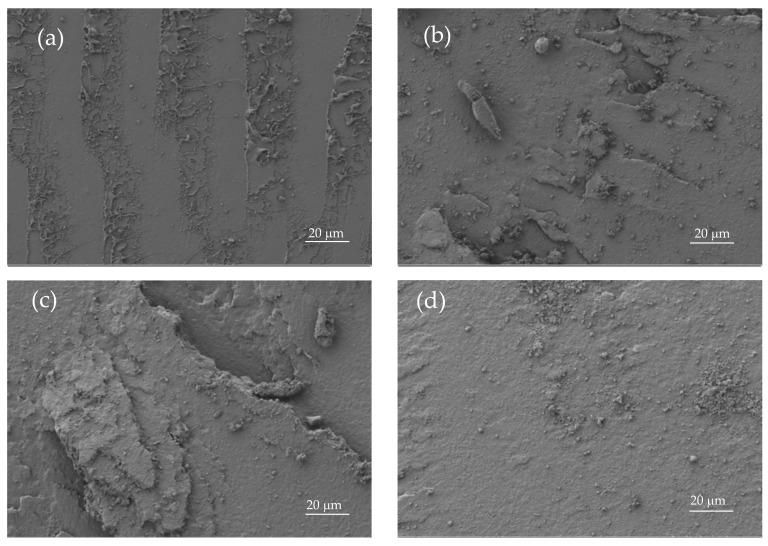

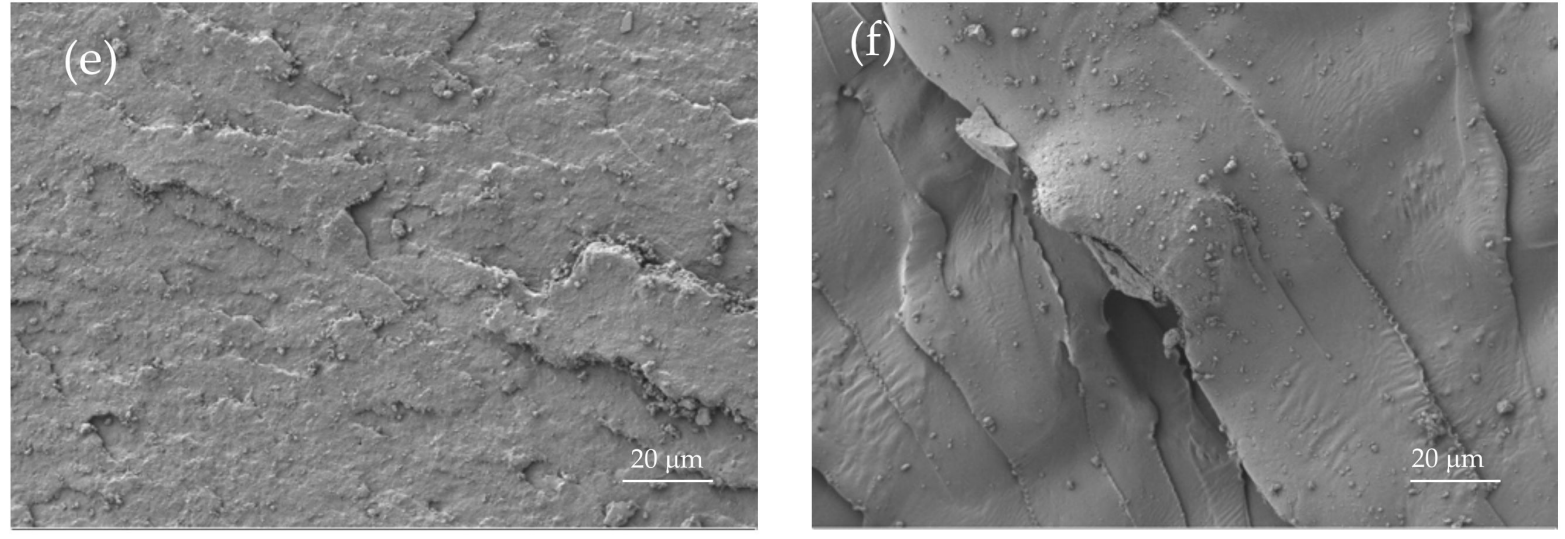
Field emission scanning electron microscopy (FESEM) images at ×500 corresponding to fractured surfaces from impact tests of (**a**) PLA, (**b**) PLA_90_PBSA_10_, (**c**) cPLA_80_PBSA_20_, (**d**) PLA_80_PBSA_20_, (**e**) PLA_70_PBSA_30_, and (**f**) PBSA_._

This particular behaviour is directly related to the morphology of the developed materials (Figure 3). Figure 3a shows the FESEM images of the fractured surfaces corresponding to unblended PLA. As it can be seen, the surface is uniform and smooth typical of a brittle fracture, with different crack growths [41]. This morphology is in total agreement with previous mechanical results. A remarkable change in the fracture surface morphology can be observed in Figure 3e, which corresponds to the binary PLA/PBSA blend with 30 wt % PBSA. The surface is not as smooth and it shows increased roughness due to increased plastic deformation. Similar morphology can be observed for uncompatibilized PLA/PBSA blends with different PBSA loading. The compatibilized blend with 20 wt % PBSA shows different fracture morphology due to its higher elongation at break, which allows more deformation before fracture (Figure 3c). It can be seen in Figure 3c that the surface presents a greater tear on it, which suggests increased PLA-PBSA interaction, because the compatibilizer works as a bridge between these two polymers, as reported Eslami et al. [45]. Obviously, PBSA shows a typical ductile fracture (Figure 3f), with a wavy surface topography that is representative for plastic deformation (even in impact conditions).

### 3.2. Thermal Behaviour of Binary PLA/PBSA Blends

**Figure 4:**
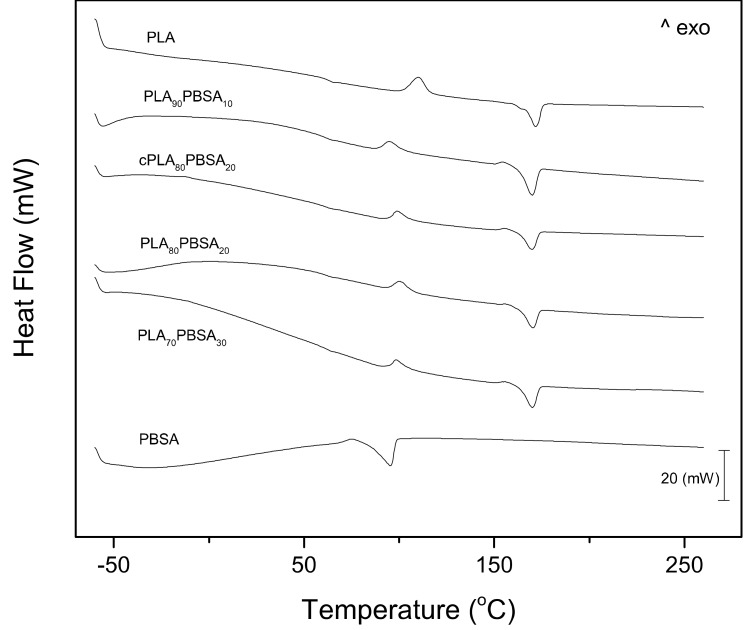
Differential scanning calorimetry (DSC) scans (second heating cycle) corresponding to binary PLA/PBSA blends.

Figure 4 shows the DSC thermograms corresponding to the second heating cycles of the developed PLA/PBSA blends, while Table 4 gathers the main parameters that were obtained by DSC. The first thermal transition that can be observed in Figure 4 is the glass transition temperature, T_g_ of the PLA-rich phase. This is located between 60–70 °C. As the temperature rises above the T_g_, an exothermal peak appears between 95–110 °C, which corresponds to the cold crystallization process of PLA chains. This cold crystallization process is related to a rearrangement of the PLA chains to an ordered structure that is activated by temperature. At higher temperatures comprised in the 155–170 °C range, an endothermic peak is observed, which is attributable to the melting process of the crystalline domains in PLA [46]. Regarding neat PBSA, it shows a melting process comprised between 70–100 °C that overlaps the cold crystallization process of PLA. Neat PLA shows a T_g_ of 63.4 °C and this is slightly reduced to 61.1 °C in the blend with 10 wt % PBSA, which suggests slight miscibility between these two poly(esters). Higher PBSA contents of 30 wt % only promote a slight decrease in T_g_ down to values of 60.6 °C, which corroborates the restricted miscibility between PLA and PBSA. This slight change in the T_g_ by the addition of PBSA to PLA is a clear indication of restricted miscibility of these two biopolymers, as reported Lee et al. [5]. Another important finding that can be outlined from DSC thermograms is the cold crystallization process. Although it overlaps with the melting process of PBSA, one important change can be identified. In particular, the cold crystallization process is moved toward lower temperatures, which means that the energy barrier for PLA crystallization is reduced. This could be due to partial miscibility between PLA and PBSA, but the most important mechanism that is responsible for this is the melting of the PBSA-rich phase that contributes to increase chain motion, thus allowing PLA chains to arrange into a packed structure at lower temperatures, due to the lubricant effect of the melted PBSA-rich phase, as reported by Lee et al. [5] in previous researches. In particular, the cold crystallization peak temperature, T_cc_ is reduced below 100 °C, while the typical T_cc_ for neat PLA is close to 109 °C. As expected, the compatibilization effect that Joncryl^®^ provides leads somewhat to a restriction in chain motion, thus leading to a slightly higher T_cc_ value, as compared to all other blends. This similar behaviour has been reported in PLA-based materials by L. Quiles-Carrillo et al. [47] in PLA/Almond shell flour composites that were compatibilized with ESAO. The T_cc_ of PLA/ASF composites that were obtained were even higher than neat PLA. This shift of the cold crystallization process indicates that crystallites of PLA can be formed at lower temperatures due to the effect of PBSA, as suggested by Ojijo et al. [39]. With regard to the melt peak temperature of PLA (T_m_PLA_), the changes are negligible. Neat PLA shows a T_m_ of 170.9 °C and the melt peak temperature of the PLA-rich phase in the binary PLA/PBSA blends decreases to 168 °C. Frenz et al. [48] have reported an increase in the melt strength of PLA and other poly(esters) by the addition of chain extenders, such as Joncryl^®^, but no remarkable changes in the peak temperature can be observed.

**Figure 5:**
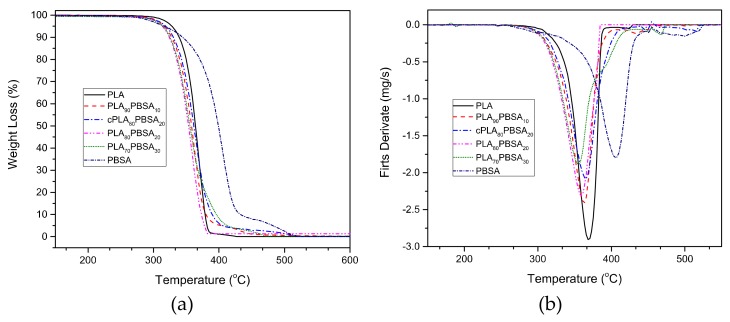
Thermogravimetric (TGA) degradation curves corresponding to binary PLA/PBSA blends, (**a**) thermogravimetry (TG) mass loss and (**b**) differential thermogravimetry (DTG) first derivative.

Regarding the thermal stability at high temperatures, Figure 5 gathers the comparative TGA degradation curves corresponding to the neat PLA, neat PBSA, and PLA/PBSA blends. The TGA thermograms indicate that neat PLA and PLA/PBSA blends degradation occurs in a single step process. Regarding neat PBSA, its degradation process takes place in two stages. The first one occurs at about 400 °C, with an associated weight loss of around 90%, and it is in accordance with Renoux et al. [38]. The second stage is about 500 °C and only a 10% weight loss occurs.

Table 5 shows the thermal parameters that are related to the different materials, specifically the characteristic temperatures at 5% weight loss (T_5%_) and the maximum degradation rate temperature (T_max_). Even though a slight decrease in the thermal stability in T_5%_ can be detected by the presence of PBSA, in general, it does not cause a noticeable effect in the PLA matrix. In fact, the PLA/PBSA blends show typical PLA behaviour. At high temperatures, a slight increase in thermal stability can be observed, the influence of PBSA on the PLA/PBSA mixtures is evident, since they show a behavior close to the PBSA at a temperature range that is close to its degradation, with neat PBSA being the most thermally stable. The compatibilization effect that Joncryl^®^ provides leads to gaining thermal stability and it can be observed in Figure 5. The uncompatilized blend containing 20 wt % PBSA degrades at earlier temperature when compared with its compatibilized counterpart, which corroborates what Frenz et al. [48] reported. 

### 3.3. Shape Memory Behaviour of Binary PLA/PBSA Blends

Several authors have proposed different techniques to characterize the shape memory behaviour in biopolymers, such as tensile and DMA tests [49,50]. These are specifically used to determine the recovery rate after subjecting the sample to a specific thermal cycle. Despite this, the use of a conventional bending test is widely used because of its simplicity and the quality of the information that it can provide. The measurement of the recovery angle is a qualitative/quantitative method to visualize the shape memory behavior and it represents a way to understand the shape memory behaviour of the blends and the effect of the PBSA addition. Table 6 shows the recovery percentage of the neat PLA and PLA/PBSA blends after the programming and recovery cycle. As we expected, the neat PLA presents high values of shape memory reaching a recovery of 70% and 58% corresponding to the 90° and 120° flexural deformation, respectively. Notice that PLA shape memory effect works better for small deformations.

**Figure 6:**
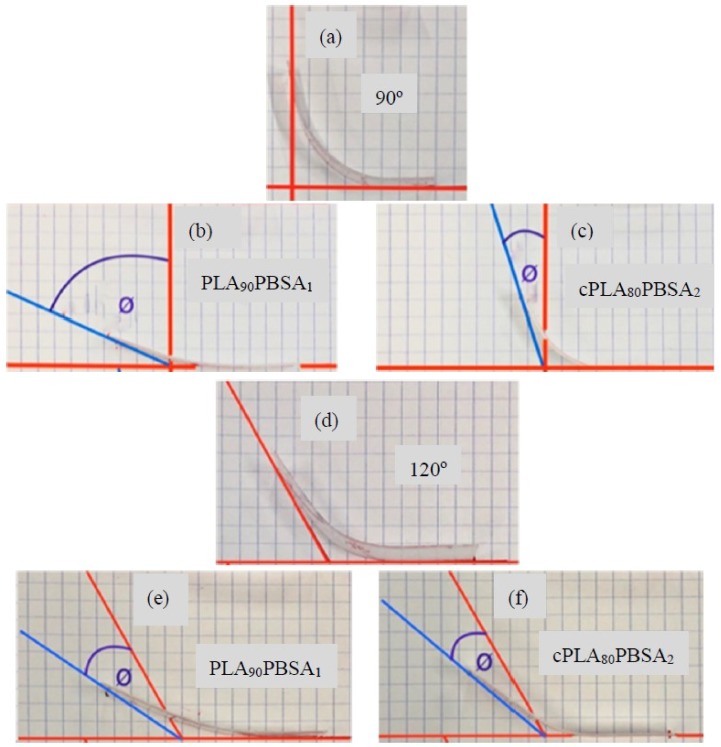
Images of the shape memory behaviour of binary PLA/PBSA blends in flexural conditions with a permanent shape after deformation and shape recovery of (**a**) temporary shape (90°), (**b**) shape recovery of PLA90PBSA10 (90°), (**c**) shape recovery of cPLA80PBSA20 (90°), (**d**) temporary shape (120°), (**e**) shape recovery of PLA90PBSA10 (120°), and (**f**) shape recovery of cPLA80PBSA20 (120°).

In fact, with the addition of PBSA, the PLA shape memory capability tends to decrease. It is worthy to note the particular case of PLA_90_PBSA_10_ shown in Figure 6b,e, which presents the best recovery behaviour of all of the developed materials, even better than the neat PLA. Tcharkhtchi et al. [38] remarked that, to get an adequate shape memory effect, the PBS percentage must be of about 20%, which gives consistency to the obtained results.

The clear negative effect that is provided by Joncryl^®^ is noticeable, since almost all of the shape memory capability of the mixtures is lost, reaching values 20% and 35%, corresponding to the 90° and 120° deformation, respectively, as can be seen in Figure 6c,f.

## 4. Conclusions

Injection molding and the hot-press molding process made binary PLA/PBSA blends. The influence of PBSA has a noticeable effect on mechanical performance, decreasing the tensile modulus and strength, which means that the brittle behaviour of the blends has diminished, thus leading to an increase in mechanical ductile properties. The compatibilized blend shows elongation at break values of 121%, remarking the evidence of the compatibilization of these two polymers, adding up to the high values that were obtained in the impact test, suggesting the improvement on toughness.

The thermal analysis shows that the presence of PBSA induces a slightly decrease in PLA glass transition temperature (T_g_), which corroborates the low miscibility between PLA and PBSA. The PBSA effect is noticeable in the PLA matrix, enhancing the PLA crystallization, and leading to a low cold crystallization temperature (T_cc_). Although the presence of PBSA induced a decrease in the shape memory behavior on PLA, 10 wt % PBSA in a PLA/PBSA blends shows the best shape memory recovery, reaching values that were even better than PLA.

## Figures and Tables

**Table 1:** Composition and labelling of PLA/PBSA binary blends.

Code	PLA (wt %)	PBSA (wt %)	Joncryl^®^ ADR (phr *)
PLA	100	-	-
PLA_90_PBSA_10_	90	10	-
cPLA_80_PBSA_20_	80	20	0.5
PLA_80_PBSA_20_	80	20	-
PLA_70_PBSA_30_	70	30	-
PBSA	-	100	-

* phr denotes the weight parts of Joncryl^®^ per hundred parts by weight of PLA/PBSA blend.

**Table 2:** Summary of mechanical properties of binary PLA/PBSA blends obtained from tensile tests.

Code	Tensile Strength, σ_T_ (MPa)	Elastic Modulus, E_T_ (MPa)	Elongation at Break, ε_b_ (%)
PLA	64.0 ± 1.2	1165 ± 44	9.2 ± 1.5
PLA_90_PBSA_10_	52.6 ± 0.8	1012 ± 21	12.2 ± 3.8
cPLA_80_PBSA_20_	41.2 ± 2.0	754 ± 47	121.2 ± 18.7
PLA_80_PBSA_20_	42.0 ± 3.6	849 ± 75	29.7 ± 6.3
PLA_70_PBSA_30_	37.7 ± 3.0	625 ± 72	56.5 ± 10.3
PBSA	18.3 ± 1.6	159 ± 61	432.7 ± 57.4

**Table 3:** Impact absorbed energy (Charpy test) and Shore D hardness of binary PLA/PBSA blends.

Code	Impact Strength (kJ m^−2^) (“V” notched)	Impact Strength (kJ m^−2^) (unnotched)	Shore D Hardness
PLA	2.48 ± 0.22	28.10 ± 2.40	78.80 ± 0.84
PLA_90_PBSA_10_	2.54 ± 0.34	23.03 ± 2.80	74.00 ± 2.74
cPLA_80_PBSA_20_	4.33 ± 0.02	28.90 ± 0.85	75.00 ± 1.00
PLA_80_PBSA_20_	3.28 ± 0.28	27.52 ± 2.13	73.00 ± 1.41
PLA_70_PBSA_30_	5.75 ± 0.60	N/B	72.20 ± 1.60
PBSA	26.02 ± 0.60	N/B	57.00 ± 0.71

**Table 4:** Main thermal parameters obtained by differential scanning calorimetry (DSC) and of binary PLA/PBSA blends.

Code	T_g_PLA_ (°C)	T_cc_PLA_ (°C)	T_m_PLA_ (°C)	T_m_PBSA_ (°C)
PLA	63.4 ± 0.6	109.9 ± 1.1	170.9 ± 3.3	-
PLA_90_PBSA_10_	61.1 ± 1.2	94.5 ± 1.7	168.9 ± 2.2	-
cPLA_80_PBSA_20_	61.1 ± 0.9	98.7 ± 1.4	168.8 ± 2.7	-
PLA_80_PBSA_20_	61.7 ± 0.6	100.3 ± 1.2	169.4 ± 2.6	-
PLA_70_PBSA_30_	60.6 ± 1.2	97.9 ± 1.0	168.7 ± 2.9	-
PBSA	-	-	-	95.2 ± 1.4

**Table 5:** Main thermal parameters obtained by thermogravimetric analysis (TGA) of binary PLA/PBSA blends.

Code	TGA		
	T_5%_ (°C)	T_Max_ (°C)	Mass_Residual_ (%)
PLA	328.7 ± 5.25	368.1 ± 6.3	0.05 ± 0.01
PLA_90_PBSA_10_	317.0 ± 4.4	362.5 ± 4.3	0.02 ± 0.02
cPLA_80_PBSA_20_	319.3 ± 3.5	365.4 ± 5.2	0.12 ± 0.02
PLA_80_PBSA_20_	312.3 ± 5.2	359.5 ± 5.4	1.37 ± 0.01
PLA_70_PBSA_30_	314.7 ± 4.7	355.8 ± 5.7	0.20 ± 0.01
PBSA	317.0 ± 5.7	405.3 ± 7.8	0.01 ± 0.01

**Table 6:** Shape memory behaviour parameters corresponding to binary PLA/PBSA blends.

Code	Permanent Shape (^o^)	Temporal Shape (^o^)	Final Shape (^o^)	Recovery (%)	Temporal Shape (^o^)	Final Shape (^o^)	Recovery (%)
PLA	180	90	153	70	120	155	58
PLA_90_PBSA_10_			155	72		158	63
cPLA_80_PBSA_20_			108	20		141	35
PLA_80_PBSA_20_			144	60		146	43
PLA_70_PBSA_30_			109	21		140	33
